# Functional Analysis of the *Arlequin* Mutant Corroborates the Essential Role of the *ARLEQUIN/TAGL1* Gene during Reproductive Development of Tomato

**DOI:** 10.1371/journal.pone.0014427

**Published:** 2010-12-23

**Authors:** Estela Giménez, Benito Pineda, Juan Capel, María Teresa Antón, Alejandro Atarés, Fernando Pérez-Martín, Begoña García-Sogo, Trinidad Angosto, Vicente Moreno, Rafael Lozano

**Affiliations:** 1 Departamento de Biología Aplicada, E. Politécnica Superior, Universidad de Almeria, Almería, Spain; 2 Instituto de Biología Molecular y Celular de Plantas (UPV-CSIC), Universidad Politécnica de Valencia, Valencia, Spain; 3 Departamento de Biología Vegetal y Ecología, E. Politécnica Superior, Universidad de Almería, Almería, Spain; Purdue University, United States of America

## Abstract

Reproductive development of higher plants comprises successive events of organ differentiation and growth which finally lead to the formation of a mature fruit. However, most of the genetic and molecular mechanisms which coordinate such developmental events are yet to be identified and characterized. *Arlequin* (*Alq*), a semi-dominant T-DNA tomato mutant showed developmental changes affecting flower and fruit ripening. Sepals were converted into fleshy organs which ripened as normal fruit organs and fruits displayed altered ripening features. Molecular characterization of the tagged gene demonstrated that it corresponded to the previously reported *TOMATO AGAMOUS-LIKE 1* (*TAGL1*) gene, the tomato ortholog of *SHATTERPROOF* MADS-box genes of *Arabidopsis thaliana*, and that the *Alq* mutation promoted a gain-of-function phenotype caused by the ectopic expression of *TAGL1*. Ectopic overexpression of *TAGL1* resulted in homeotic alterations affecting floral organ identity that were similar to but stronger than those observed in *Alq* mutant plants. Interestingly, *TAGL1* RNAi plants yielded tomato fruits which were unable to ripen. They displayed a yellow-orange color and stiffness appearance which are in accordance with reduced lycopene and ethylene levels, respectively. Moreover, pericarp cells of *TAGL1* RNAi fruits showed altered cellular and structural properties which correlated to both decreased expression of genes regulating cell division and lignin biosynthesis. Over-expression of *TAGL1* is able to rescue the non-ripening phenotype of *rin* and *nor* mutants, which is mediated by the transcriptional activation of several ripening genes. Our results demonstrated that *TAGL1* participates in the genetic control of flower and fruit development of tomato plants. Furthermore, gene silencing and over-expression experiments demonstrated that the fruit ripening process requires the regulatory activity of *TAGL1*. Therefore, *TAGL1* could act as a linking factor connecting successive stages of reproductive development, from flower development to fruit maturation, allowing this complex process to be carried out successfully.

## Introduction

Reproductive development of higher plants entails a succession of developmental steps, from floral bud generation to fruit ripening and seed dispersal, all aimed at ensuring progeny survival. Such biological processes are finely controlled by different transcription factors, most of which belong to the MADS-box family [1], [2]. Extensive genetic and molecular studies performed in several model plant species have led to a broadly accepted model of flower development based on the combinatory activity of three gene functions which determine floral organ identity, i.e. the ABC model [3], [4]. More recently, new regulatory functions have been added and a revised model based on the formation of MADS protein complexes has been proposed [5]. In tomato (*Solanum lycopersicum* L.), A- and C-class genes are represented by *MACROCALYX* (*MC*; [6]) and *TOMATO AGAMOUS1* (*TAG1*; [7]), while *Le-DEFICIENS* (*Le-DEF;* [8]) is considered a B-class gene. Among other floral functions, *MC* is involved in the development of sepals in the first whorl, whereas *TAG1* specifies carpel identity of fourth whorl organ primordia. In addition, *TAG1* seems to participate in fruit development, as deduced from its expression pattern and the phenotypes shown by plants where *TAG1* has been either overexpressed or inhibited [7], [9].

As in most flowering plants, fruit development of tomato begins with ovary fecundation and goes through three phases [10]. The earliest one takes place around flower anthesis and involves the development of the carpels forming the ovary and the decision to proceed with fruit development or to abort. During the second phase, the fruit grows due primarily to cell division and the embryos start their development. Accordingly, genes regulating cell division [11], [12] and cell cycle [13], [14] are highly expressed in developing fruits. Cell division ceases at the third phase and fruit growth continues by cell expansion until the fruit achieves its final size [15]. Subsequently, increases in the respiration rate and ethylene synthesis occur in fully developed fruits allowing their ripening. Accordingly, tomato *ACS* and *ACO* genes [16] and ethylene receptor genes, mainly *NR* [17], [18] and *ETR4* [19], [20] are activated during fruit ripening. Furthermore, the genetic and physiological characterization of tomato ripening mutants, *ripening-inhibitor* (*rin*; [21]), *non-ripening* (*nor;* [22]) and *Colorless non-ripening* (*Cnr*; [23]), together with the molecular isolation of the mutated genes, have demonstrated that other important regulatory factors must be properly coordinated with the ethylene signal to carry out the ripening program. *RIN* [6], *NOR* [24] and *CNR* [25] genes encode transcription factors belonging to the MADS-box, NAC-domain and SBP-box families, respectively. They act upstream of ethylene biosynthesis and are key functions for the genetic control of fruit ripening [26]. Interestingly, RIN [27] and two regulatory proteins recently reported as involved in fruit ripening, the TAGL1 MADS-box factor [28], [29] and the HB-1 homeobox protein [30], are able to bind to the promoter region of *ACS2* [27], [29] and *ACO1* [30] genes, respectively, proving that transcriptional factors directly regulate the activity of ethylene biosynthesis genes in tomato.

Besides the regulatory pathways, studies concerning fruit ripening in tomato have also focused on the biochemical and physiological changes taking place during the ripening process, such as chlorophyll degradation, sugar and pigment accumulation, production of aroma and flavour components, cell wall metabolism and softening [31]–[33]. Examples of the best characterized ripening genes include those encoding the fruit specific polygalacturonase (PG) and pectinesterase (PE), two enzymes involved in cell wall degradation associated to fruit softening [34], [35], as well as, phytoene synthase (PSY), responsible for the synthesis of lycopene, the red pigment characterizing ripe tomatoes [36]. However, recent studies show that fruit softening is not only a consequence of cell metabolism; biomechanical properties of fruit pericarp are also important cues which regulate fruit development [37], [38]. Thus, the cuticle *per se* functions as an external structural element that adds mechanical support for tissue integrity [39]. Also, peroxidase-mediated stiffening of fruit cell walls has been hypothesized as a control mechanism by which cell expansion within the fruit mesocarp, and hence fruit growth, is regulated [40]. It has also been suggested that peroxidase isozymes may restrict fruit expansion through their involvement in the lignification process [41]. Lignin has been considered a necessary component for dry fruit ripening as the lignification of valve margin cells adjacent to the dehiscence zone contributes to pod shatter [42]. This process is accurately regulated by the redundant *SHATTERPROOF1* (*SHP1*) and *SHP2* genes as well as by *FRUITFUL* (*FUL*), all of which are MADS-box genes [2], [43], the latter acting as a negative spatial regulator of the *SHP* genes. They regulate valve separation mediated by the formation and lignification of the dehiscent zone [44]. As in dry fruits, lignification of pericarp also occurs in fleshy fruits [45], indeed *SHP* and *FUL* homologues have been described in peach suggesting their implication in modulating properties of lignified endocarp of fleshy fruits [46]. The lack of mutants in this and other fleshy-fruited species has hindered thorough studies on the genetic and molecular mechanisms underlying developmental differences between dry and fleshy fruits. Indeed, few tomato genes have been isolated with important roles in the functional processes of the reproductive development such as carpel differentiation, fruit setting, fruit growth and ripening [6], [7], [25], [28], [30].

In this work, molecular and functional characterization of *Arlequin* (*Alq*), a new tomato T-DNA mutant, has allowed us to clone the tagged gene, which is *TAGL1,* a MADS-box member of the tomato *AGAMOUS-like* family previously reported [9]. Expression and functional analyses have supported evidence that *TAGL1* regulates different processes of reproductive development in tomato which involve the identity and development of carpels and the ripening of fruits. Therefore, *TAGL1* could act by connecting different sequential steps leading to the formation of a ripe tomato fruit. In this developmental scenario, the functional role of *TAGL1* also requires the participation of other ripening regulators such as *RIN*, *NOR* and *CNR.*

## Results

### The *Alq* insertional mutation affects reproductive development of tomato plants

**Figure 1:**
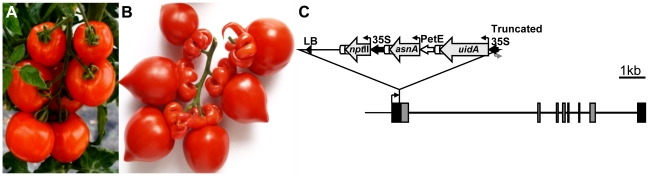
Phenotype and molecular characterization of the *Arlequin* mutant. Mature fruits from wild type (A) and *Alq* mutant (B) plants, the latter showing ripening sepals. (C) Genomic organization of the *TAGL1* gene and the T-DNA insertion in the *Alq* mutant. *TAGL1* exons are depicted as grey (coding sequence) and black (5′ and 3′ non-coding sequences) boxes. Known promoter sequence is represented by a thin line and intron sequences as solid lines. The T-DNA insertion contains the left border (LB) and three genes (*nptII*, coding for neomycin phosphotransferase II; *asnA* coding for asparagine sinthetase; *uidA,* coding for β-glucuronidase) located in reverse orientation to *TAGL1*. These genes are controlled by the 35S promoter of the cauliflower mosaic virus (35S), the pea plastocyanin promoter (*petE*) and a truncated 35S promoter, respectively. Origins of the transcription are represented as broken arrows. Scale bar in panel C: 1 kb.

The *Arlequin* (*Alq*) mutant was isolated from the screening of T-DNA mutant collections generated by using different binary vectors, the most common one included an enhancer-trapping construct [47]. The *Alq* mutation affected flower development, particularly the identity of sepal organs (first floral whorl) which were converted into carpels (normally developed in the fourth floral whorl). Both the epidermal cell morphology and the presence of trichomes and stomata on the *Alq* sepal surface were characteristics to that occurring during carpel development. Such homeotic changes lead to the development of succulent organs which grow and ripen like a normal tomato fruit (Figure 1A, B), while other reproductive or vegetative features were not altered in *Alq* plants. Genetic analysis performed on T1 and T2 progenies confirmed that the *Alq* mutant phenotype was inherited as a monogenic and semi-dominant trait. Southern blot hybridizations demonstrated that *Alq* mutant plants carried a single copy of the T-DNA [47], which in turn co-segregated with the *Alq* mutation (see Figure S1), indicating that the tagged gene was responsible for the mutant phenotype.

### Cloning and molecular characterization of the *TAGL1* gene

The gene affected by the *Alq* mutation was isolated using a TAIL-PCR protocol [48]. This allowed the amplification and cloning of genomic regions flanking the T-DNA, which was inserted 103 bp upstream of the traslation start codon of the tagged gene, interrupting the promoter region (Figure 1C). During the insertional process the T-DNA underwent some rearrangements since the right border was removed and the 35S promoter that controls the *uid*A reporter gene was truncated (Figure 1C). Genomic sequence of the isolated gene was 10.2 kb size and was organized in eight exons and seven introns, the first exon including 374 bp of the 5′-untraslated region (GenBank Accession Number GU371906). The coding sequence displayed a complete homology with the *TOMATO AGAMOUS-LIKE1* (*TAGL1*), a MADS-box gene previously reported by Busi et al. [9]. The isolated gene encoded a protein of 269 amino acids which shows 71% similarity with the *Arabidopsis* SHATTERPROOF1 protein [2].

**Figure 2:**
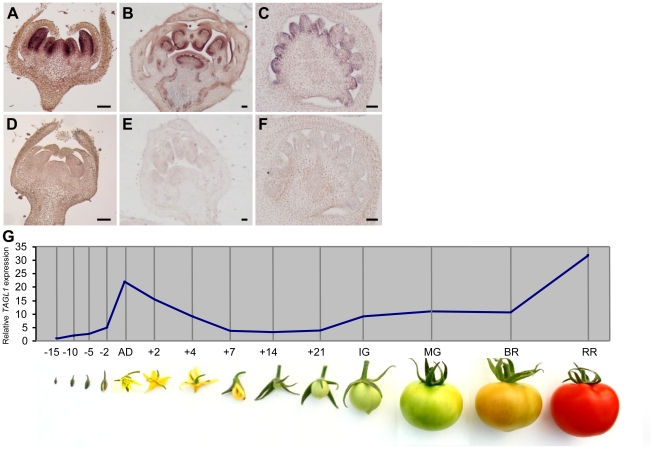
Expression of *TAGL1* during flower and fruit development. *In situ* hybridization analysis of the *TAGL1* gene in floral buds at several developmental stages (according to Brukhin et al., [48]): stage 5 (A, D), stage 8 (B, E) and stage 9 (C, F). Tissue sections were hybridized with an *TAGL1* antisense probe (A, B, C) or a sense probe (D, E, F). *TAGL1* expression during flower and fruit development was analysed by quantitative real-time PCR (G) from flowers collected 15, 10, 5 and 2 days before anthesis day (AD) and 2, 4, 7, 14 and 21 post-anthesis day. Expression in 2 cm immature green (IG), mature green (MG), breaker (BR) and ripe red (RR) fruits were also analyzed. Scale bars: 100 µm.

**Figure 3:**
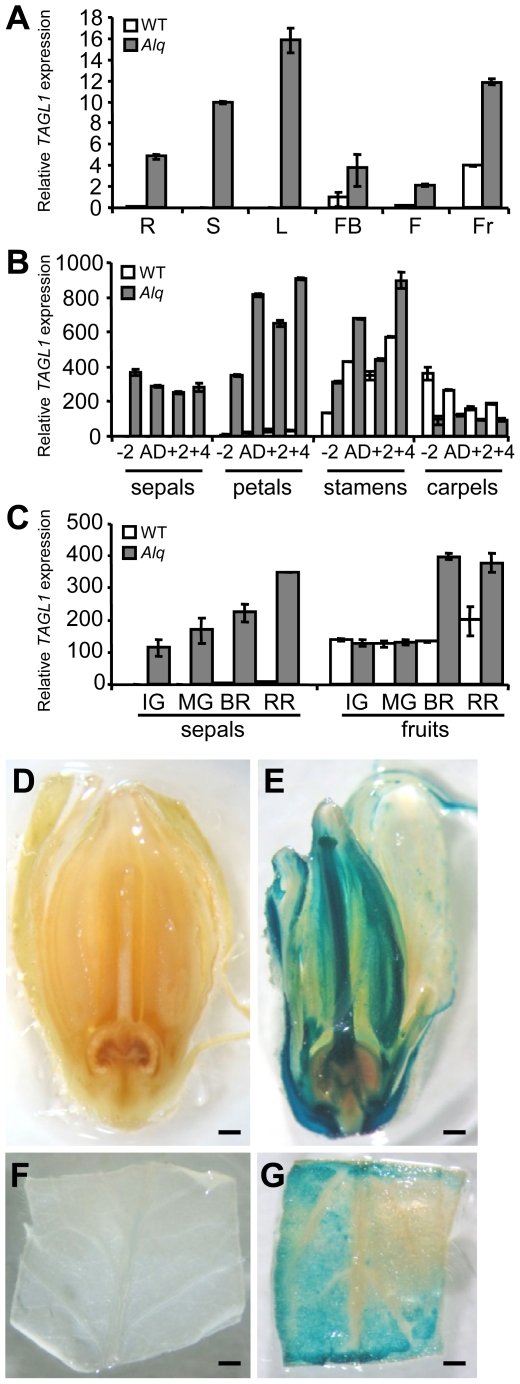
*TAGL1* expression in different plant tissues of wild-type and *Alq* mutant plants. (A) Relative quantitative RT-PCR expression analysis of the *TAGL1* gene in different plant tissues. R: roots, S: stem, L: leaves, FB: floral buds (stages 2–4), F: flowers (anthesis day), Fr: fruit (red ripe). (B) Expression of *TAGL1* gene in separate floral organs of developing flowers collected 2 days before anthesis (−2), anthesis day (AD) and 2 (+2) or 4 (+4) days post-anthesis. (C) *TAGL1* expression in sepal and fruit organs at several stages of fruit ripening, i.e. immature green (IG), mature green (MG), breaker (BR) and ripe red (RR) stages. GUS staining after histochemical *uidA* gene detection in flowers (D, E) and leaves (F, G) of wild-type (D, F) and *Alq* mutant (E, G) plants. Scale bar: 1 mm.

Spatial and temporal expression patterns of *TAGL1* were analyzed by in situ hybridization and quantitative RT-PCR experiments. Both in wild-type and *Alq* mutant plants, the *TAGL1* gene is expressed early in the two inner whorls of floral buds (stage 5 according to Brukhin et al. [49]), where stamen and carpel primordia were initiated (Figure 2A, D). Later, *TAGL1* transcripts were detectable in the endothecium tissue of anthers as well as in the ovules, placenta and vascular tissues of carpels at stage 8–9 of flower development (Figure 2B, C, E, F). A time-course experiment which included fourteen stages of flower and fruit development was performed to analyze the temporal expression pattern of the *TAGL1* gene during reproductive development (Figure 2G). Results confirmed that expression of *TAGL1* begins at early stages of flower development (Figure 2A–F) although the highest accumulation of transcripts was detected at flower anthesis and when the fruits achieved the red ripe stage (RR), i.e. 8 days after breaker stage (BR+8) (Figure 2G). Interestingly, the *TAGL1* expression was not repressed in *Alq* mutant plants as it could be expected given the molecular characteristics of the T-DNA insertion. On the contrary, a significant accumulation of *TAGL1* transcripts was detected in vegetative and reproductive organs of the *Alq* plants (Figure 3A). *TAGL1* is up-regulated in all floral organs as well as in succulent sepals and fruits at BR and RR stages of *Alq* mutant (Figure 3B, C). Such results indicate that the *TAGL1* gene is ectopically expressed in *Alq* mutant plants and promotes the homeotic conversion of sepals into fleshy fruit organs which expand and ripen as normal tomato fruits.

To elucidate the molecular nature of the gain-of-function phenotype showed by *Alq* mutant plants, we performed RT-PCR experiments using a gene specific primer of *TAGL1* and different primers designed from the truncated 35S promoter sequence present in the T-DNA insert. As a result, cDNA fragments which included part of the 35S promoter and the full length coding sequence of *TAGL1* were amplified, indicating the formation of a chimeric mRNA whose transcription started in this truncated 35S promoter. As the 35S promoter is inversely oriented with respect to the *TAGL1* coding sequence in the *Alq* mutant, it is used both to initiate the transcription of the *GUS* reporter gene (Figure 3D–G) and to control the ectopic transcription of the *TAGL1* gene, the latter in the opposite direction to the former (Figure 1C).

### Tomato plants overexpressing *TAGL1* showed a similar but stronger phenotype than *Alq* mutant plants

**Figure 4:**
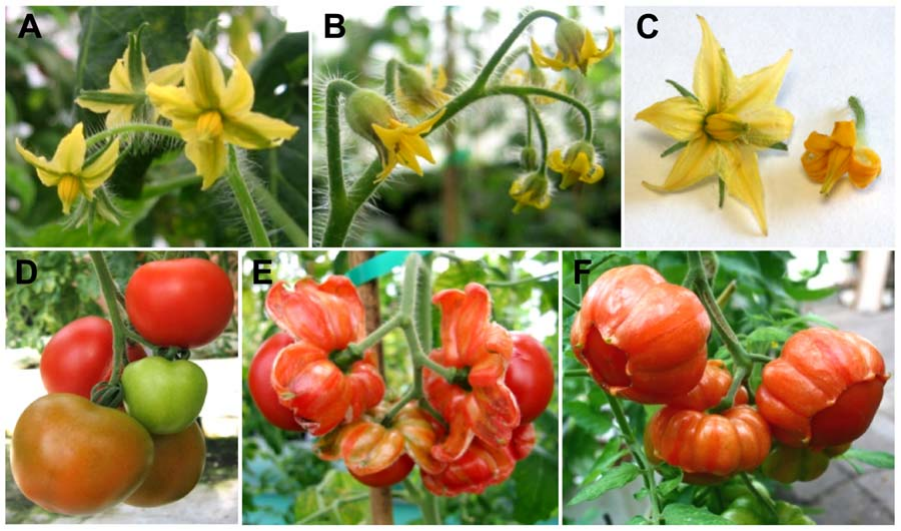
Phenotype of flowers and fruits developed by *TAGL1* overexpressing plants. Flowers (A–C) and fruits (D–F) from wild-type (A, C left and D) and 35S:*TAGL1* (B, C right, E and F) plants. Compared to wild-type plants (A, C-left and D), ectopic expression of *TAGL1* promotes visible changes affecting flowers, mainly a more intense colour and smaller size of floral organs (B, C-right). Later in the development, sepals accompanying tomato fruits show an extreme phenotype characterized by the conversion into succulent fruit organs which ripen normally (E and F).

*Alq* phenotypes suggest that ectopic expression of *TAGL1* might be responsible for the observed sepal to carpel conversions. We therefore generate transgenic tomato plants overexpressing *TAGL1* cDNA in two different genetic backgrounds, i.e. the cultivar Moneymaker (88 lines) and breeding line SLDG2 (11 lines) by using a constitutive 35S promoter gene construct. For comparative analyses, homozygous T1 and T2 transgenic lines were selected by PCR assays followed by progeny tests. Phenotypic differences were not observed between backgrounds; most 35S:*TAGL1* lines showed severe changes in flower development, even more extreme than those described in the *Alq* mutant plants. At anthesis stage, flowers of transgenic plants developed shorter sepals which remained laterally fused along their full length (Figure 4A, B). Petals were thicker and showed staminoid appearance; also, they changed the normal yellow pigmentation by orange and their edges were curled towards the abaxial surface (Figure 4A–C). Apparently, stamens and carpels were normally developed although the former were orange instead of yellow in color (Figure 4C). Upon fruit setting, 35S:*TAGL1* sepals were converted into expanded and succulent organs that finally ripened as a normal fleshy fruit (Figure 4D–F). In fact, they accumulated sugars (glucose and fructose), carotenoids and lycopene, which agrees with the climateric biosynthesis of ethylene occurring in these transformed organs (Table 1). All these biochemical compounds are considered characteristic to ripening fruits and were never detected in normal sepals.

**Table 1:** Physiological characterization of sepals and fruits at BR+8 stage (RR in wild-type) of plants overexpressing (35S:*TAGL1*) or silencing (*TAGL1* RNAi) the *TAGL1* gene.

Physiological trait	WT		35S:*TAGL1*		*TAGL1* RNAi	
	Sepal	Fruit	Sepal	Fruit	Sepal	Fruit
Glucose (mg/g FW)	0.15±0.05	4.69±0.31	3.28±0.31	7.12±0.44	0.15±0.01	3.01±0.22
Fructose (mg/g FW)	0.20±0.09	4.33±0.31	4.46±0.29	6.38±0.55	0.14±0.03	2.52±0.30
Soluble solids (°Brix)	0.20±0.03	4.95±0.20	4.36±0.05	7.45±0.35	0.05±0.00	3.00±0.15
Total carotenoids (µg/g FW)	0.10±0.01	22.58±3.33	23.20±4.03	26.26±4.63	0.11±0.01	9,73±1.22
Lycopene (µg/g FW)	n.d.	20.55±4.33	23.00±5.10	22.59±2.69	n.d.	1,52±0.30
Ethylene (nl/gxh)	n.d.	8.49±1.21	54.47±10.13	22,35±4.35	n.d.	1.32±0.33

Values represent mean±standard errors for a minimum of 30 samples analyzed in each genotype (10 plants and 3–4 fruits per plant). n.d. = non detected.

**Figure 5:**
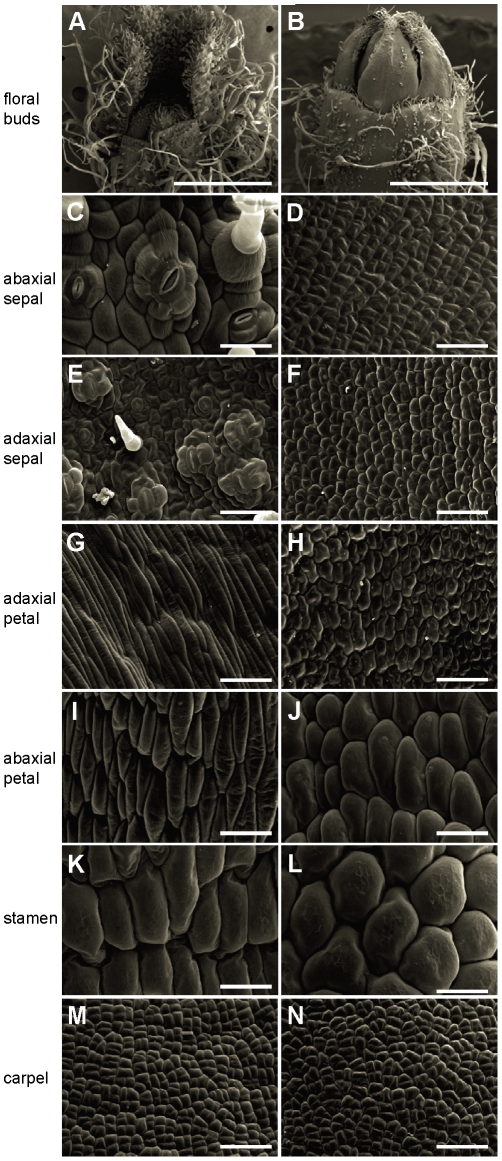
Homeotic conversion of sepals into carpels promoted by the ectopic expression of *TAGL1*. Morphological features of floral buds (A, B) and epidermal cells of floral organs (C–N) analysed by scanning electron microscopy in WT (left) and 35S:*TAGL1* (right) plants. Cell surface of 35S:*TAGL1* sepals shows similar developmental characteristics to that of wild-type carpels. Scale bars: 1 mm in A–B; 50 µm in C–N.

Scanning electron microscope analyses showed homeotic changes affecting sepal and petal development of 35S:*TAGL1* plants (Figure 5). Both on the abaxial and adaxial surfaces, epidermal cells covering sepal primordia displayed small size and regular morphology resembling those forming wild-type carpel epidermis (Figure 5C–F, M, N). Moreover, stomata and long hairs, whose presence is characteristic of normal sepals, were absent in 35S:*TAGL1* floral buds (Figure 5C–F). Similarly, epidermal cells on the adaxial surface of young petals showed carpel-like features, mainly rounded shape and random disposition, while at abaxial surface they were almost identical in morphology and size to stamen cells (Figure 5G–N). No homeotic changes were observed in the innermost whorls (stamen and carpel) of the 35S:*TAGL1* flowers (Figure 5K–N). Therefore, changes of cell identity promoted by the ectopic expression of the *TAGL1* gene in sepals and petals should be responsible for the homeotic transformations observed in 35S:*TAGL1* plants. It is noteworthy that all identity changes observed in tomato plants overexpressing *TAGL1* gene are coincident to those observed in *Alq* mutant plants, indicating that their phenotype is promoted by the ectopic expression of *TAGL1*.

### *TAGL1* silencing lines were altered in reproductive development and fruit ripening

**Figure 6:**
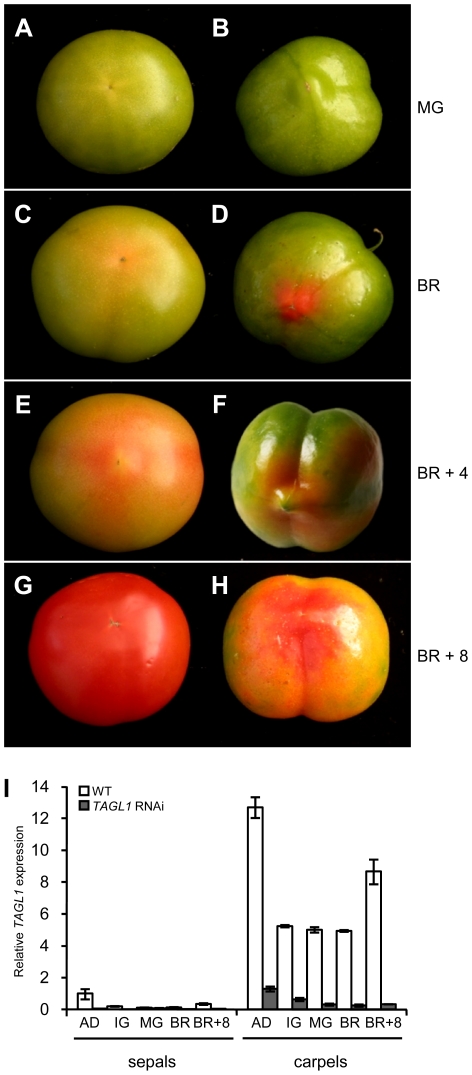
Phenotypic and gene expression analyses of *TAGL1* silenced fruits (RNAi lines). Tomato fruits were collected at mature green (MG, panels A, B), breaker (BR, panels C, D), BR+4 (E, F) and BR+8 (G, H) from wild-type (A, C, E, G) and RNAi (B, D, F, H) plants. (I) Expression of *TAGL1* in sepals and carpels of wild-type (WT) and *TAGL1* RNAi plants at several stages of fruit development: anthesis day (AD), immature green (IG), mature green (MG), breaker (BR) and BR+8.

With a view to analyze the functional role of the *TAGL1* gene in greater depth we generated independent *TAGL1* silencing lines using an interference RNA approach (RNAi). RNAi lines were also obtained in the cv. Moneymaker (77 lines) and the SLDG2 line (27 lines), being the observed phenotypes of T1–T2 homozygous plants quite similar in both genetic backgrounds. As revealed by phenotypic and SEM analyses, there were no floral organ identity changes either in floral buds or mature flowers produced by RNAi plants, despite the fact that expression levels of *TAGL1* were significantly diminished up to the basal *TAGL1* expression quantified in vegetative organs of wild-type plants (Figure 6K). However, loss-of-function of *TAGL1* gave rise to visible alterations during fruit development and ripening (Figure 6A–J), while it did not affect sepal development. At mature green (MG) stage, RNAi tomatoes showed more intense green color and a shinier and rougher surface than wild-type fruits (Figure 6A, B). Later in development, the ripening process was initiated but RNAi fruits never reach the red pigmentation and softening appearance which characterize wild-type fruits (Figure 6C–H). Instead, they were of a pale yellow-orange colour and stiffer appearance (Figure 6G–H), which is also maintained several weeks later. At biochemical level, chlorophyll content was higher in MG fruits of RNAi plants, which agrees with their darker green color. Also sugars, carotenoids, and particularly lycopene, were accumulated at lower levels in RNAi ripening fruits (Table 1).

Given the regulatory function of ethylene as activator of climateric ripening of fleshy fruits, we analyzed whether the non-ripening features characterizing *TAGL1* silenced fruits could be associated to changes in ethylene biosynthesis. Thus, levels of ethylene measured by gas chromatography were significantly lower than those of wild-type ones, indicating that transcriptional activity of *TAGL1* is required for fruit ripening mediated by ethylene (Table 1). According to this result, qPCR experiments demonstrated that the climacteric increase of *TAGL1* expression associated to fruit ripening did not occurred in RNAi fruits (Figure 6K).

### *TAGL1* influences expression patterns of tomato genes involved in reproductive development and fruit ripening

**Figure 7:**
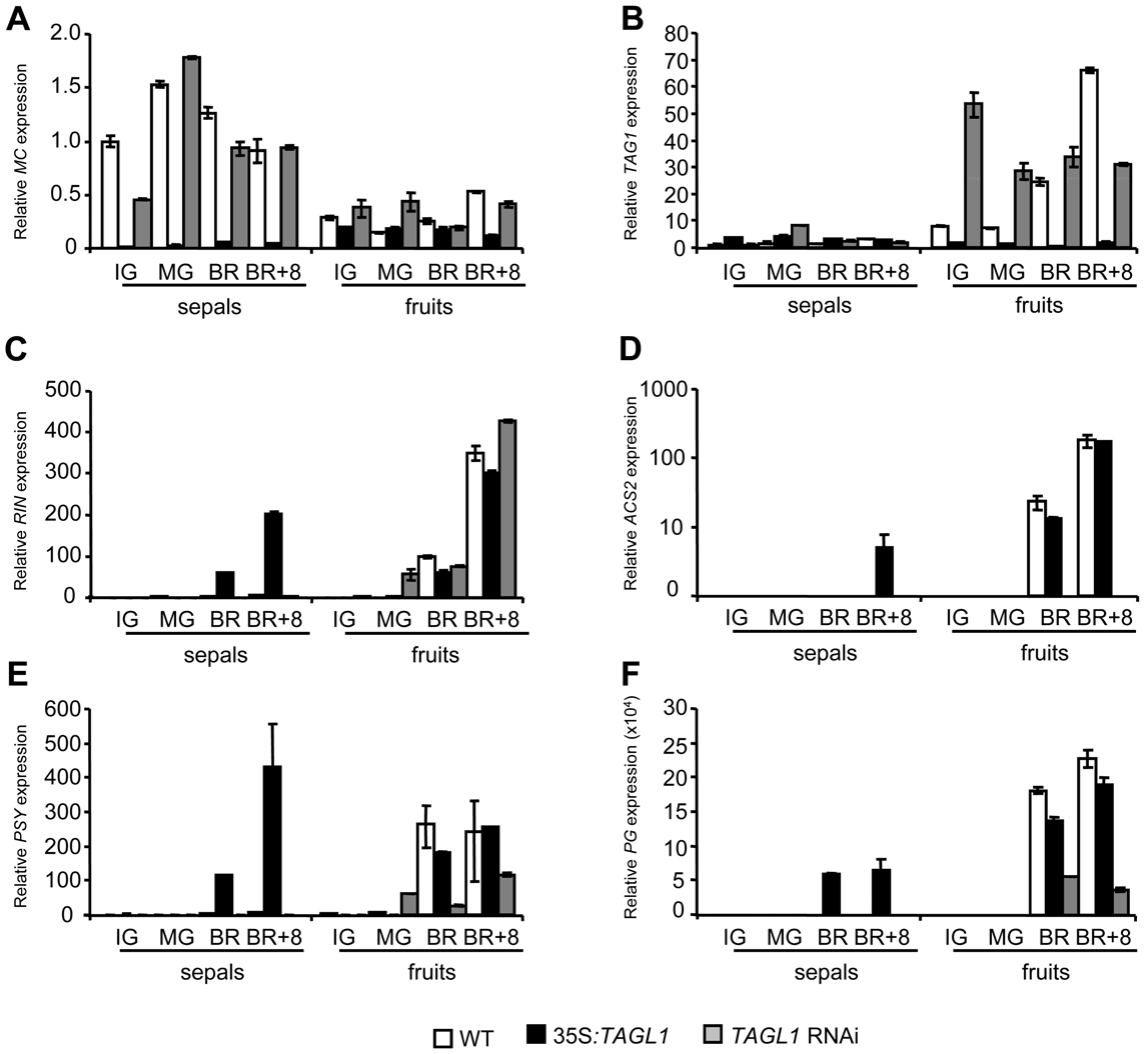
*TAGL1* influences expression of genes involved in flower development and fruit ripening. Relative quantitative RT-PCR analyses of *MC* (A), *TAG1* (B), *RIN* (C), *ACS2* (D), *PSY* (E) and *PG* (F) genes in sepals and fruits of wild-type (WT), 35S:*TAGL1* and *TAGL1* RNAi plants at immature green (IG), mature green (MG), breaker (BR) and BR+8 stages of fruit ripening.

Comparative expression analyses were carried out either in overexpressing or silencing *TAGL1* lines in order to analyze genetic interactions among *TAGL1* and other tomato genes involved in reproductive development and fruit ripening of tomato (Figure 7; see Table S1). Given the homeotic alterations observed during flower development of 35S:*TAGL1* plants, expression level of floral organ identity genes representative of A-, B- and C- class MADS-box genes were analyzed. The A-class *MC* gene [6], which confers sepal identity to the first whorl organ primordial, was down-regulated in transformed sepals of 35S:*TAGL1* plants. Such inhibition was detected during fruit development but not at flower anthesis. Expression of *MC* was not altered in *TAGL1* RNAi fruits suggesting that factors other than *TAGL1* may regulate *MC* activity (Figure 7A). Expression of the B-class *Le-DEF* gene [8] was not modified as result of changes in *TAGL1* expression, neither in sepal nor in fruit organs (see Table S1). The *TAG1* gene specifies stamen and carpel identity in tomato flowers [7] and is considered the most closely related gene to *TAGL1* [28], [50]. Transcription level of *TAG1* was not altered during floral development of plants overexpressing *TAGL1* but was notably repressed during fruit ripening. Accordingly, *TAG1* was up-regulated during the same developmental stages of *TAGL1* RNAi lines suggesting compensatory mechanisms of gene expression between these two paralogous genes (Figure 7B) and demonstrating the specificity of the gene construct employed to silence ALQ.

Likewise, transcription level of several set of genes involved in the development and ripening of tomato fruit was analyzed by RT-qPCR experiments. Respect to wild-type plants and with independence of the reproductive organ considered (floral or fruit organ), significant differences in the expression levels of *TAGL11* and *TDR4* genes, all required for fruit development, were not detected in 35S:*TAGL1* nor in RNAi plants (see Table S1). The only exception was the higher expression of *TDR4* whose transcripts were slightly accumulated in ripened sepals of 35S:*TAGL1* plants probably due to the involvement of *TDR4* in fruit ripening [33].

Taking into account the climacteric nature of fleshy tomato fruits, expression levels of genes involved in the ethylene synthesis and perception pathways *ACO1*, *ACS2*, *ACS4, NR, RIN* and *NOR* [24], were analyzed during fruit ripening stages (Figure 7C, D; see Table S1). Transcripts of all these genes were accumulated in transformed sepals of 35S:*TAGL1* plants to levels quite similar to those observed in ripening fruits. Silencing of *TAGL1* resulted in no expression changes of most genes mentioned above, with the singular exception of *ACS2*, which is significantly repressed in RNAi ripening fruits (Figure 7D).

Phenotypic analyses of transgenic plants either silencing or overexpressing *TAGL1* revealed changes in the expression patterns of genes involved in the final steps of fruit ripening, particularly those regulating carotenoid biosynthesis and cell wall degradation. Thus, expression analysis of *PSY1, PG*, *PE2* and *E4* [33], [51] genes were down-regulated in tomato fruits of RNAi plants, which is congruent with their yellow-orange color and stiffness appearance. As expected, these genes were markedly up-regulated in succulent sepals of 35S:*TAGL1* plants, a feature never observed in wild-type sepals (Figure 7E, F; see Table S1).

### *TAGL1* overexpression rescues the phenotype of non-ripening tomato mutants

**Figure 8:**
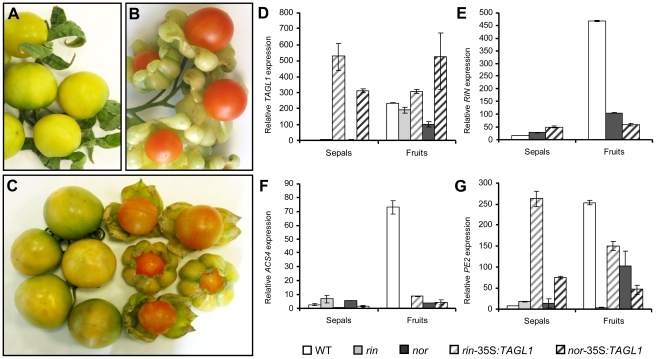
Overexpression of *TAGL1* rescues the phenotype of *rin* and *nor* ripening mutants. The non-ripening phenotype of fruits yielded by *rin* (A) and *nor* (C-left side) mutant plants is partially restored by over-expression of the *TAGL1* gene as shown the phenotype of *rin*-35S:*TAGL1* (B) and *nor*-35S:*TAGL1* (C-right side) transgenic lines. Relative quantitative RT-PCR analyses of the ripening genes *TAGL1* (D), *RIN* (E), *ACS4* (F) and *PE2* (G) performed either in the wild-type background (cv. Ailsa Craig), *rin, nor*, *rin*-*35S:TAGL1* and *nor*-*35S:TAGL1* sepals and fruits at BR+8 ripening stage.

To gain further insight into the functional role of the *TAGL1* gene in fruit ripening, we checked whether constitutive expression of *TAGL1* was sufficient to rescue the phenotype of non-ripening mutants *rin* and *nor*. We generated transgenic plants by overexpressing *TAGL1* cDNA in *rin* (8 independent lines) and *nor* (10 independent lines) mutant backgrounds. Tomato fruits yielded by most of these transgenic lines (5 *rin*-35S:*TAGL1* lines and 7 *nor*-35S:*TAGL1* lines) rescued the ripening phenotype, i.e. they displayed red pigmentation, softening, and developed fleshy fused sepals (Figure 8A–C). The restored phenotype was mendelian inherited by selfing progenies, as expected. Subsequent expression analyses of ripening genes (Figure 8; see Table S2) demonstrated that, compared to the wild-type background (cv. Ailsa Craig), *TAGL1* expression was not altered either by *rin* or *nor* mutations (Figure 8D). Similarly, constitutive expression of *TAGL1* in *rin* and *nor* mutants fruits did not change *TAG1, TDR4*, *RIN* (Figure 8E) and *NOR* transcript levels at BR+8 stage (Table S2), indicating that transcriptional factors encoded by these ripening genes are not regulated by *TAGL1*. However, expression of *ACS2* and *ACS4*, as well as of *PSY*, *PG*, *PE2* and *E4*, increased with respect to the corresponding mutant backgrounds (Figure 8F, G, Table S2), which agreed with the ripening phenotype restored by *TAGL1* (Figure 8A–C). Indeed, *PE2* and *E4* reached transcription levels similar to those shown by the wild-type background (cv. Ailsa Craig), particularly at mature green stage (data not shown). Expression of *ACO1* was also up-regulated when *TAGL1* was overexpressed in *rin* fruits but not in *nor* fruits, and the opposite occurred with *CNR* suggesting differences in the *ACO1* and *CNR* regulation by *RIN* and *NOR* genes.

Despite to *rin* and *nor* mutant fruits overexpressing *TAGL1* develop fleshy sepals, they were able to initiate the ripening process as suggested their orange color (Figure 8A–C). This observation differ from the less extreme phenotype described by Itkin et al. [29], most likely due to expression differences of the transgen. However, a higher accumulation of *NOR*, *CNR*, *PE2*, *PSY*, *ACO1* and *ACS2* transcripts was detected in *rin*-35S:*TAGL1* sepals, although the transcript levels never achieved those detected in wild-type fruits (see Table S2). Likewise, *RIN, CNR, PE2, PSY*, *E4* and, to a lesser extent, *ACS2* genes were up-regulated in *nor*-35S:*TAGL1* sepals. These results support that *TAGL1* is necessary but not sufficient to fully activate fruit ripening in sepals, a process which also required the contribution of *RIN* and *NOR*.

It is worthy to note that the rescued phenotype shown by *rin*-35S:*TAGL1* plants was stronger than those described by Itkin et al. [29], which may be due to differences in the cDNA sequence of *TAGL1* that these authors overexpressed. This sequence corresponded to the unigene SGN-U581068 (http://solgenomics.net/), which contained three point mutations leading to two amino acid changes in the encoded protein (Gly216Ans and Phe243Ser).

### Cellular and structural characteristics of *TAGL1* silenced tomato fruits

**Figure 9:**
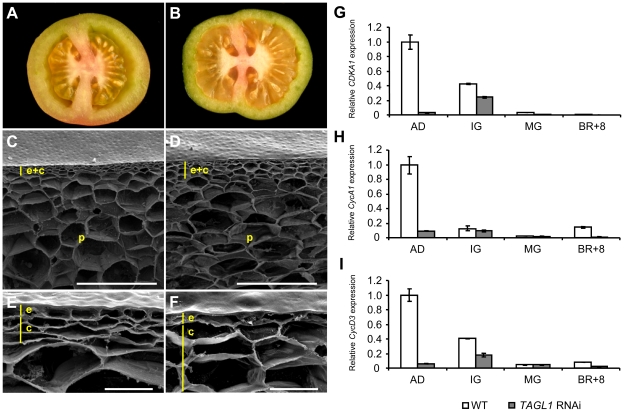
Fruit development of *TAGL1* RNAi tomato lines. Transversal sections of WT (A) and *TAGL1* RNAi (B) fruits. Morphological features of epidermal (e), collenchyma (c), and parenchyma (p) cells observed in the pericarp of WT (C) and *TAGL1* RNAi (D) fruits by scanning electron microscopy. A detailed view of the different cell types is also shown (E–F). Expression analyses of cell cycle related genes show decreased transcript levels of *CDKA1* (G), *CycA1* (H) and *CycD3* (I) in RNAi pericarp at early stage of fruit development. Several stages fruit development and ripening were analyzed: anthesis day (AD), inmature green (IG), mature green (MG) and breaker+8 (BR+8). Vertical yellow lines indicate epidermal (e) and collenchyma (c) cell layers. Scale bar: 500 µm in C–D; 50 µm in E–F.

**Figure 10:**
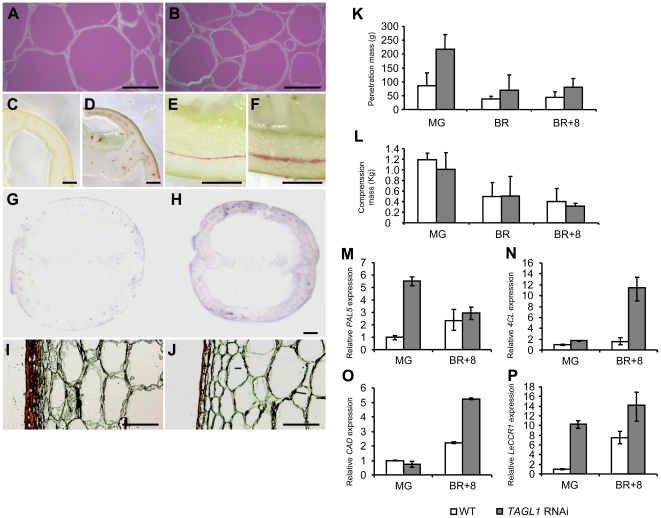
Structural and cellular properties of *TAGL1* silencing fruits. Calcofluor White staining of cellulose in paraffin-embedded sections of pericarp from ripen wild-type (A) and *TAGL1-*silenced (B) fruits. Phloroglucinol staining of lignin in transversal (C, D) and longitudinal (E, F) sections of pericarp from WT (C, E) and RNAi (D, F) fruits. Peroxidase activity in tissue prints of WT (G) and *TAGL1-*silenced (H) red fruits. Sudan III staining of pericarp sections from WT (I) and RNAi (J) fruits. (K) Penetration test of intact tomato fruit from WT and RNAi plants. (L) Compression analysis of fruit pericarp from WT and RNAi plants. In K and L the fruits were tested at mature green (MG), breaker (BR) and BR+8 stages. (M–P) Expression analysis of lignin biosynthesis genes (*PAL5, 4CL, LeCCR1* and *CAD*) performed in WT and RNAi fruits from mature green (MG) and breaker+8 (BR+8) stages. Scale bar: 50 µm in A–B; 5 mm in C–H; 50 µm in I–J.

*TAGL1* repressed tomatoes display a pale orange color and stiffness appearance (Figure 6G, H) and, most remarkably, a visible reduction of pericarp thickness, from around 50% in MG to 25% in RR stages (Figure 9A, B). At ripening stage, both epidermal and subjacent collenchyma cells of wild-type fruit pericarp showed similar size (Figure 9C, E), however, the latter were significantly enlarged (up to 4-fold) in the pericarp of *TAGL1* RNAi fruits (Figure 9D, F), as scanning electron microscopy confirmed. On the contrary, smaller parenchyma cells and greater intercellular spaces were observed in these fruits, indicating failures of cell adhesion and expansion in this fruit compartment (Figure 10A, B) promoted by *TAGL1* silencing. The higher size of collenchyma cells was correlated to a decreased cell number per cell surface unit. Similarly, a reduction in the number of parenchyma cell layers was observed by Vrebalov et al. [28] in the fruit pericarp of *TAGL1* repressed plants. Taken together, these observations suggest alterations in the cell division pattern promoted by silencing of *TAGL1* in pericarp tissues. To check this hypothesis, we analyzed the expression levels of tomato genes regulating cell cycle, in particular *CDKA1*, *CycA1* and *CycD3* [13], [14]. To check this hypothesis, we analyzed the expression levels of tomato genes regulating cell cycle, in particular *CDKA1*, *CycA1* and *CycD3* [13], [14]. Interestingly, all of them were down-regulated in *TAGL1* RNAi developing fruits (Figure 9G–I).

*TAGL1* RNAi phenotypes also suggested alterations in the pericarp structure which required a more detailed analysis. Thus, pericarp tissue at MG, BR and BR+8 stages was subjected to two types of mechanical assays, i.e. compression and penetration. Independently of the ripening stage analyzed, RNAi fruits displayed almost similar compression firmness but increased resistance to penetration (Figure 10K, L), indicating textural or compositional differences of pericarp cells. In agreement to phenotypic observations, one third reduction of swelling capacity was detected in cell walls of RNAi pericarp at BR+8 stage (see Figure S2). Also, examination of fracture planes confirmed some differences in cell wall properties since the regular morphology and integrity of RNAi pericarp cells were conserved from MG to B+8 stages, a feature never observed in wild-type fruits (see Figure S2). The lower swelling capacity and the higher cell integrity of RNAi pericarp at B+8 stage (when wild-type fruits are fully ripe) resembled to those showed by wild-type pericarp at MG stage and agree with the increased stiffness and stronger appearance showed by *TAGL1* silenced fruits.

Recent results have involved to the peroxidase activity on the cell wall stiffness of tomato fruit skin, a function which is mediated by its participation in lignin biosynthesis [40], [41]. Thus, peroxidase activity and lignin content were analyzed to elucidate plausible causes of stiffness and alterations displayed by cell walls of RNAi pericarps. While peroxidase activity is restricted to epidermal cells and radial vascular network of normal tomato fruits, it is widely detected in all pericarp tissues, including parenchyma tissue, of *TAGL1* silenced fruits (Figure 10G, H). Accordingly, lignin content was significantly higher in RNAi fruits, where 2.5-fold increase in lignin thioglycolic acid (LTGA) content and a greater number of vascular tissues were observed, the latter being thicker than in wild-type fruits (Figure 10C–F). Taking into account these results, expression of the tomato genes *LeCCR1, CAD, 4CL* and *PAL*, all involved in lignin biosynthesis [52], [53] were analysed at MG and BR+8 stages of fruit ripening. Results obtained showed increased transcript levels of *LeCCR1, CAD* and *4CL* genes in RNAi fruits at BR+8 stage (RR in the wild-type background). Indeed, *LeCCR1* and *PAL* were up-regulated at previous stages, i.e. at MG stage (Figure 10M–P).

Together with the structural characteristics of fruit pericarp, the cuticle plays an important role as an external non-cell structure which adds biomechanical support and cooperates for tissue integrity of ripening fleshy fruits [38], [54]. Therefore, we performed a comparative analysis of cuticle between wild-type and RNAi fruits at BR+8 stage. While the former developed a substantial epidermal cell encasement (11,46±0,24 µm thickness), the latter displayed a thinner cuticle (4,02±0,15 µm thickness; p<0.001, n = 30) covering the outer epidermal cell layer, which in turn was unable to invaginate through the inner epidermal cell layers (Figure 10I, J).

## Discussion

Genetic, molecular and functional analyses of the *Alq* T-DNA mutant have allowed us to characterize the *TAGL1* gene as a key MADS-box regulator of reproductive development in tomato thus proving the importance of insertional mutant collections as useful tools for functional genomics studies in tomato [55]–[57]. Instead of being a null allele, *Alq* mutant allele promoted the ectopic expression of the *TAGL1* gene both in vegetative and reproductive organs. However, the gain-of-function phenotype of the *Alq* mutant was mainly observed in sepals, which were homeotically converted into carpel-like organs that in turn ripen as normal fruit organs. The rearrangement suffered by the T-DNA during the integration process placed a truncated 35S promoter in reverse orientation to the tagged gene. This promoter was used by the cellular machinery to activate the genes located both in forward (*uidA*) and reverse (*TAGL1*) orientation. These serendipity results demonstrated the usefulness of activation tagging approaches to identify plant genes with redundant functions or lacking obvious loss-of-function alleles in tomato [58].

### *TAGL1* participates in the genetic control of reproductive development of tomato

MADS-box genes were found to play central roles in flower and fruit development of angiosperms [1], [2], [6], [28], [59]. The MADS-box *TAGL1* is expressed during early stages of flower development and its transcripts was detected in stamen and carpel organs. However, the highest expression level of *TAGL1* occurred at flower anthesis and later, at the onset of fruit ripening, indicating that its function is required during the whole reproductive development of tomato. Constitutive expression of *TAGL1* promotes developmental conversion of sepals and petals into carpel-like and staminoid organs, respectively. Similar homeotic changes were described in tomato plants overexpressing *TAG1*, a C-class MADS-box gene involved in stamen and carpel development [7]. Nevertheless, *TAG1* is not expressed in sepals of 35S:*TAGL1* flowers indicating that *TAGL1* is capable of specifying reproductive identity to perianth organ whorls even in the absence of *TAG1*. These results also suggest that *TAGL1* and *TAG1* could act redundantly during reproductive development of tomato plants. Unexpectedly, homeotic changes affecting floral organ identity were not observed in *TAGL1* silencing lines while conversion of stamens and carpels into petals and sepals, respectively, was described for plants expressing an antisense *TAG1* construct [7]. Most likely, the lack of *TAGL1* expression is compensated by *TAG1* since its mRNA level increases in *TAGL1* RNAi flowers (Figure 7). Moreover, *TAG1* and *TAGL1* genes overlap in their expression domains and displayed similar temporal expression patterns. Together, these results indicate that *TAGL1* and *TAG1* should play overlapping regulatory functions as genetic determinants of stamen and carpel development, which may be the result of balanced expression patterns of both genes. Likewise, overexpression of *SHP1* and *AG*, the orthologues to *TAGL1* [28], [50] and *TAG1* [7] respectively, in *Arabidopsis*, also promoted the development of flowers with carpelloid sepals and staminoid petals [2], [60]. Furthermore, *SHP* and *AG* genes play overlapping roles regarding carpel identity, and *SHP1* has even retained the ability to substitute *AG* activity in stamens [59].

Development of tomato flowers also required that *TAGL1* expression is excluded from sepal and petal primordia whose organ identities depend on the activity of A- and B-class genes. In fact, the tomato A-class gene *MC* is normally expressed in wild-type sepals, where *TAGL1* is repressed; however, *MC* transcripts were not detected in transformed sepals of *TAGL1* overexpressing plants. Such behaviour suggests functional similarities between tomato *MC* and *TAGL1* genes and *Arabidopsis AP1* and *AG* genes, since the antagonist roles of the latter ones are needed for the appropriate development of sepals and carpels in the first and fourth floral whorl of *Arabidopsis*, respectively [61]. These results support that homeotic genes encoding MADS-box transcriptional factors have conserved most of the regulatory functions required for flower development in different plant species [62], [63].

After flower anthesis, *TAGL1* expression increases during fruit development of wild-type plants. Silencing of *TAGL1* in transgenic fruits promotes developmental alterations of fruit pericarp, similar to those described by Vrebalov et al. [28], mainly reduced thickness and changes in the number and size of collenchyma cell layers. Furthermore, swelling of cell walls and cell adhesion, which are characteristic features of normal tomato fruits, are also altered in pericarp tissues of RNAi fruits. Such abnormalities were observed even before fruit ripening was initiated and demonstrate that *TAGL1* is involved in tomato fruit development. Similarly, *SHP1*, the orthologous of *TAGL1* in *Arabidopsis*, also regulated fruit development [2]. However, constitutive expression of *TAGL1* seemed not to be completely sufficient to rescue the normal shattering of *shp1 shp2* double mutant *Arabidopsis* suggesting functional divergences between *TAGL1* and *SHP1* [28]. These are most likely related to the different types of fruit produced by tomato and *Arabidopsis*, i.e. fleshy berries and dry siliques, respectively.

### *TAGL1* plays an essential role as positive regulator of fruit ripening

As fleshy and climateric fruits, ripening of tomato fruits involved hormonal, genetic and physiological changes some of which depend on ethylene synthesis while others are regulated by independent-ethylene pathways [24], [26], [64], [65]. Ectopic expression of *TAGL1* not only promotes the homeotic conversion of sepals to carpelloid organs but also their ulterior ripening as normal tomato fruit organs, which agree to the results previously described by Vrebalov et al. [28] and Itkin et al. [29]. In addition, our results proved that this ectopic ripening of sepals is caused by the activation of the ripening pathway promoted by *TAGL1*, which is capable to induce the expression of *CNR, NOR, RIN* and *TDR4* genes. Subsequently, the enhanced expression of *ACS2*, *ACS4* and *ACO1* would explain the increased levels of total carotenoids, lycopene, sugars and ethylene observed in those fleshy organs. On the contrary, *TAGL1* silenced lines fail to complete fruit ripening likely due to the reduced *ACS2* expression and hence, the lower ethylene synthesis. Consequently, expression of genes encoding enzymes involved in pigment accumulation, *PSY*, and cell wall degradation, *PG* and *PE2*, were down-regulated in RNAi fruits. These results indicate that *TAGL1* regulates tomato fruit ripening through an ethylene-dependent pathway, although the relationships between *TAGL1* and other transcriptional factors controlling fruit ripening requires a greater consideration.

Among the transcription factors involved in the ethylene-mediated ripening pathway, those encoded by *RIN* and *NOR* genes seem to be essential in this process as they act upstream to the ethylene genes [6], [21]. To investigate the hierarchical relationships of *TAGL1* with the ripening genes, expression analyses on genotypes bearing different allele combinations and expression levels of *RIN, NOR* and *TAGL1* genes were performed. Our results showed that expression of *RIN, NOR* and *CNR* was not modified by silencing *TAGL1* nor was *TAGL1* expression changed in the *rin* and *nor* mutants. Furthermore, the ripening process is activated by *TAGL1* even in the absence of *RIN* and *NOR* functions as demonstrated not only the rescued phenotypes showed by *rin-* and *nor-*35S:*TAGL1* plants but also the increased expression of genes involved in ethylene synthesis (e.g. *ACS2* and *ACS4*) and cell wall metabolism (e.g. *PG* and *PE2*). Therefore, these results support that *TAGL1* regulates fruit ripening through an ethylene pathway independent to that of *RIN* and *NOR*. Both regulatory pathways seem to converge in *ACS2* as deduced from the lower transcript levels of this gene detected in *rin*, *nor* and *TAGL1* silencing genotypes. Ripening activity promoted by *ACS2* could depend on the genetic interaction between *RIN* and *TAGL1*. The formation of RIN-TAGL1 heterodimers revealed by two-hybrid experiments [66], and the capacity of RIN [27] and TAGL1 [29] to bind *ACS2* promoter support this hypothesis.

When expressed in *rin* and *nor* mutant plants *TAGL1* is able to rescue the ripening phenotype of fruits (Figure 8). These observations provide further evidence that not only *RIN* and *NOR* but also *TAGL1* regulates for fruit ripening, most likely by activating *ACS2*, *ACS4* and *PSY*, *PG*, *PE2* and *E4.* However, ectopic expression of *TAGL1* in *rin* and *nor* mutant plants was able to rescue the ripening phenotype of fruits but not of succulent sepals, suggesting that other fruit-specific factors rather than *TAGL1* might operate independently to *RIN* and *NOR* to promote fruit ripening.

Besides the transcription factors mentioned above, other regulatory genes have been involved in reproductive development of tomato [24], [67]. Indeed, protein interactions involving ripening transcription factors as well as the capacity of the latter to bind ethylene-related gene promoters have recently been reported in tomato [9], [27], [30], [66]. Several studies have demonstrated that flower development is achieved by the formation of large MADS protein complexes [1], [68]. Therefore, it is reasonable to postulate that ALQ, RIN, NOR, CNR and other ripening proteins may function together in one or more transcriptional complexes through which ripening of fleshy fruits could be regulated.

### Ripening control mediated by *TAGL1* includes structural and cell properties of fruit pericarp

During fruit development, cell division activity is mainly focused on outermost layers of pericarp [10]. We detected a reduced number of collenchyma cells in the fruit pericarp when *TAGL1* expression is inhibited, and also Vrebalov et al. [28] found a decreased number of parenchyma cell layers in *TAGL1* RNA fruits. Such observations are likely due to a decreased cell division activity as suggested the lower expression of tomato genes regulating cell cycle *CDKA1, CycA1* and *CycD3* [13], [14]. Furthermore, the smaller size of parenchyma cells placed just below the collenchyma tissue suggests that cell expansion has not been fully achieved. Both decreased cell division and cell expansion could explain the reduced pericarp thickness showed by *TAGL1* silenced fruits and prove the regulatory function of *TAGL1* as positive regulator of fruit development.

Maturation of fleshy fruits entails disassembly of cell walls and changes in polysaccharide composition, which are also accompanied by textural changes of pericarp tissues [24]. Repression of the *TAGL1* gene promotes decreased expression level of genes associated to cell wall degradation, which could explain the stiffness of tomato fruits (measured by a penetration test). Most importantly, stiffer cell walls of ALQ RNAi fruits also contain higher amounts of lignin indicating modified compositional and textural properties of fruit pericarp. Accordingly, expression of genes involved in lignin biosynthesis was up-regulated and peroxidase activity increased in *TAGL1* repressed fruits. Important roles have been attributed to peroxidase during lignification of plant tissues, among others it is thought to mediate changes in the mechanical properties and stiffness of exocarp cell wall [40], [41]. The greater peroxidase activity and lignin content are likely to be responsible for changes affecting cell wall stiffness and expansion of fruits yielded by *TAGL1* silencing plants. Together, these results indicate that *TAGL1* could regulate fruit ripening in part through the control of the lignification process occurring in pericarp cells of tomato fruits.

Compositional changes of the cell wall affect softening and texture of ripening fruits, but equivalent alterations in the cuticle development also influence their biochemical and structural features. Therefore, both disassembly of cell wall and cuticle architecture should be regulated as part of the fruit ripening program of fleshy fruits [38]. Ripening fruits lacking *TAGL1* expression showed a significant reduction of cuticle thickness and lack of cuticle invaginations among the epidermal cells. Such abnormalities could be related to the reduced number of epidermal cells from which cuticle is formed and suggest a narrow relationship between the cuticle development and the non-ripening phenotype of *TAGL1* silenced fruits. In addition, cell morphology and turgor, which also contribute to textural features of ripening fruits [69], seem to be influenced by cuticle development [38], [54]. We detected loss of intercellular adhesion and altered cell morphology of pericarp tissues when *TAGL1* is repressed. On the other hand, Vrebalov et al. [28] observed higher water loss and more rapid dehydration in *TAGL1* RNAi fruits, which might be directly influenced by the thinner cuticle they developed, as we have reported. These results involve the cuticle development as a modulating factor of fruit ripening regulated by the *TAGL1* gene. Further analyses are however required to weigh up the importance of structural, compositional and biomechanical characteristics of cuticle during this developmental process.

### Conserved developmental functions in dry and fleshy fruits

*TAGL1* gene plays a crucial role as part of the gene network which controls fruit ripening of tomato plants, as has been previously reported [28], [29]. Furthermore, this work provides a detailed study about the genetic functions of *TAGL1* during flower and fruit development of tomato. This study started from the cloning and characterization of the *Arlequin*, a semi-dominant mutant allele of *TAGL1* gene. Therefore, bearing in mind the availability of the *Alq* mutant phenotype and the results and conclusions here reported, we propose the name *ARLEQUIN* (*ALQ)* for the previously reported *TAGL1* gene.

Recently, a discrete number of regulatory genes encoding transcription factors required for fruit ripening have isolated. Among them, *RIN* [6], *NOR* [24], *CNR* [25] and *HB-1* [30] seem to regulate ethylene-related genes although their hierarchical relationships are not fully known. We also support evidence that *ALQ*/*TAGL1* also acts upstream to ethylene-related genes though independently to the ripening pathway regulated by *RIN*. All these transcription factors participate together in the ripening control of fleshy fruits, however, *ALQ*/*TAGL1* also regulates flower and fruit development and therefore, cannot be considered as a specific fruit ripening gene. Instead, *ALQ*/*TAGL1* might act as a linking factor between flower development and fruit ripening networks, allowing the reproductive development to be successfully completed. The homology and putative redundancy between *ALQ*/*TAGL1* and other floral organ genes, such as *TAG1*, support the idea that some floral MADS-box genes could have evolved by acquiring novel fruit ripening functions during angiosperm evolution as also happens with *AG* and *SHP1* genes of *Arabidopsis* [59].

In addition, *ALQ*/*TAGL1* seems to control structural features of fruit pericarp. *ALQ*/*TAGL1* repression promotes an elevated peroxidase activity associated to a greater lignification of pericarp tissues, the latter is likely to be due to the increased expression of lignin biosynthesis genes. As consequence, *ALQ*/*TAGL1* silenced tomato fruits loses in some extent their fleshy appearance for acquiring a ligneous and hardness one (Figure 6). It is known that distinct types of fruits differ in the lignification degree of pericarp tissue, which in turn is analogous to the valve tissue of *Arabidopsis* silique [45]. Although lignified endocarp cells have been observed in both siliques and fleshy fruits [43], [45], [70], lignification is absolutely needed for dehiscence of dry siliques as developed by *Arabidopsis*. In this species, *SHP1* regulates differentiation of the dehiscence zone allowing the lignification of adjacent cells and the subsequent shattering of valves [2]. Similarly, *ALQ*/*TAGL1* seems to regulate lignin biosynthesis allowing fleshiness of tomato fruits though the genetic network involved in this regulatory pathway remains yet unknown. Considering the evolutionary origin of fleshy fruits [71], the function of *ALQ*/*TAGL1* regulating structural features of tomato fruits could have evolved from that existing in dry-fruited related species [71]. Together, these results provide further evidence that genetic and physiological mechanisms underlying fruit ripening control are conserved between dry and fleshy fruits. It does not exclude that singular functions are also required to regulate specific ripening pathways in each type of fruits. This is the case of *RIN*, *NOR*, *CNR* and *HB1* genes in tomato [6], [25], [30], [72].

## Materials and Methods

### Plant material

The tomato (*Solanum lycopersicum*) mutant *Arlequin* and its genetic background, a breeding line named SLDG2, have been described elsewhere [47]. The cultivar Moneymaker, the ripening mutants, *rin* and *nor*, and their genetic background Ailsa Craig, were provided by C.M. Rick Tomato Genetics Resource Center (http://tgrc.ucdavis.edu/). Plants were grown under greenhouse conditions using standard practices with regular addition of fertilizers.

### DNA isolation and Tail-PCR

Genomic DNA was isolated from young leaves using Plant DNAzol Reagent (Invitrogen). Sequences flanking the *Alq* insertion were amplified by thermal asymmetric interlaced PCR (TAIL-PCR) as described by Liu et al. [48]. The *uid*A sequence specific primers GUS1, GUS2 and GUS3 (Table S3) were used whereas the AD primers have been previously described [48], [73].

### GUS staining assays

Fluorimetric assays were performed as described by Jefferson et al. [74]. Samples were incubated overnight at 37°C in a solution of 2 mM 5-bromo-4-chloro-3-indolyl glucuronide (Sigma). GUS activity was examined after extraction of chlorophyll with 70% ethanol and observed under binocular lens. Assays were repeated at least twice.

### Generation of *TAGL1* transgenic tomato plants

The *TAGL1* complete open reading frame was amplified from *S. lycopersicum* cv. Moneymaker cDNA using primers 35SALQF (Table S3) to introduce a *Bam*HI site in the 5′ untranslated leader of *TAGL1* cDNA and 35SALQR that introduced a *Kpn*I site in the 3′ untranslated sequence. The PCR product was cloned and sequenced. The resulting plasmid was digested with *Bam*HI and *Kpn*I, and the *TAGL1* cDNA was subcloned into the binary vector pROKII [75] to generate an overexpression (35S:*TAGL1*) gene construct.

In order to down regulate expression of the *TAGL1* gene, an interference RNA (RNAi) approach was followed. A 298 bp fragment of the *TAGL1* cDNA was amplified using primers RNAiALQF to introduce a *Xba*I and a *Xho*I site and RNAiALQR to introduce a *Cla*I and a *Kpn*I sited and cloned into pGEM-T easy to create plasmid ALQ2. The insert of ALQ2 was liberated by *Xho*I and *Kpn*I digestion, and cloned into vector pKannibal [76] to generate plasmid pKannibal-ALQ. Plasmid ALQ2 was digested with *Xba*I and *Cla*I and the restriction fragment was cloned in pKannibal-ALQ to obtain plasmid ALQ-RNAi. The resulting plasmid was digested with *Not*I and the entire construct was cloned into the binary vector pART27 [77] to express inverted repeat sequences of *TAGL1* separated by intronic sequences under the control of the constitutive promoter 35S.

The generated binary plasmids were electroporated into *Agrobacterium tumefaciens* LBA 4404 strain for further use in genetic transformation experiments. *Agrobacterium*-mediated transformation of cotyledons from seedlings was performed following the protocols described by Ellul et al. [78].

T2 generations were obtained from *TAGL1* RNAi and 35S:*TAGL1* transgenic plants to compare homozygous and azygous lines, the latter used as control. Only plants homozygous for the transgenes were used for structural, biochemical and gene expression analyses.

### RNA preparation and gene expression analyses

Biological replicates of total RNA were obtained from floral organs and fruit pericarp using the Trizol reagent (Invitrogen). Contaminating DNA was removed using the DNA-free™ kit (Ambion) and 500 ng RNA was used for cDNA synthesis with a ML-MLV reverse transcriptase (Invitrogen) and a mixture of random hexamer primers and oligo-dT (18 mer) primer.

Specific primer pairs for each gene (Table S4) were used for expression analysis by real time PCR performed with the SYBR Green PCR Master Mix kit (Applied Biosystems, Foster City, CA, USA) using the 7300 Real-Time PCR System (Applied Biosystems). Data collection and analysis were performed using System Sequence Detection Software v1.2 (Applied Biosystem). Results were expressed using ΔΔCt calculation method in arbitrary units by comparison to a data point from the wild type samples. The housekeeping gene *Ubiquitine3* was used as a control in all gene expression analyses. The absence of genomic DNA contamination in the RT-PCR assays was demonstrated using an *TAGL1* promoter specific amplicon as control.

For in situ hybridization experiments, tissue preparation, sectioning and transcript detection were performed as described by Lozano et al. [79]. Antisense transcripts were synthesized using the DIG RNA labeling mix (Roche). As a negative control, sense RNA probes were hybridized with the same sections and no signals were observed under the hybridization and detection conditions used.

### Scanning-electron microscopy (SEM)

SEM studies were performed as previously described by Lozano et al. [79]. In all cases plant material was fixed in FAEG and stored in 70% ethanol. The samples were dehydrated, critical point dried with liquid CO_2_ in a critical point drier Bal Tec (Liechtenstein) CPD 030 and gold coated in a Sputter Coater (Bal-Tec SCD005). The samples were visualized with a Hitachi (Tokyo, Japan) S-3500N scanning electron microscope at 10 kV.

### Ethylene production

Four fruits from each genotype were weighed and placed in 2.6 L air-tight containers for 2 h, withdrawing 1 ml head space gas and injecting it to a gas chromatograph (Varian 3900, Palo Alto, CA, USA) fitted with a Porapak Q column and a flame ionization detector. The detector and injector were operated at 200°C and 170°C respectively, whereas oven temperature was 50°C. The flow rates of nitrogen (carrier gas), hydrogen and air were 32, 26, and 400 mL m^−1^ respectively.

### Analysis of biochemical and mechanical properties of tomato fruits

The quantity of total soluble solids was measured using a hand refractometer (Atago, Tokyo, Japan) and expressed as the refraction index in Brix degrees. Soluble sugar content was determined as described by Klann et al. [80] by chromatography on Sugar-Pack I column (300×6.5 mm) and detected with a refractive index detector (Waters 410, Milford, MA, USA). Concentrations were calculated from peak heights by comparative analysis with glucose, fructose and sucrose standards (Sigma).

Total carotenoid content of the pericarp was measured as previously described by Soto-Zamora et al. [81]. Lycopene content was measured as described by Ronen et al. [82] with minor modifications. Lycopene was separated by reverse-phase HPLC using a Delta-Pack column (C18, 5 µm, 3.9 mm×150 mm). Samples of 50 µl of methanol-dissolved pigments were injected to a Perkin-Elmer 250 binary LC pump. The mobile phase consisted of TBME (solvent A) and methanol (solvent B), which were used in a linear gradient between A and B for 30 min, at a flow of 1 ml min^−1^. The absorbance was determined at 450 nm using a Perkin-Elmer (Waltham, MA, USA) LC290 UV-Vis detector Lycopene were identified by its characteristic absorption spectra and its typical retention time compared to standard commercial compound (Sigma-Aldrich). Peak areas were integrated by the Totalchrom chromatography software (Perkin-Elmer).

Lignin quantitative assay was performed by derivatization with thioglycolic acid [83] from 25 mg of alcohol-insoluble residues (AIRs) of tomato pericarp. The AIRs were obtained from 2 g of fresh weight of green tomato pericarp following the protocols described by Fornalé et al. [84].

Staining for peroxidase activity was performed following the protocols described by Eriksson et al. [85]. For lignin analysis, transversal sections of pericarps were stained for 2 min in a 2% phloroglucinol solution in 95% ethanol, and then photographed in 37% hydrochloric acid. For cytochemical staining of cellulose, sections were treated with a solution Calcofluor White Stain (Fluka), and washed with distilled water. Sections were observed using a UV-fluorescence microscope. Cuticle was detected in 10 µm pericarp sections staining with Sudan III solution (0.2% Sudan III in 70% ethanol) for 20 min, then washed in distilled water and observed using an optic microscope.

To test cell wall properties, cubes of red tomato fruit pericarp (1 cm^3^) were frozen in liquid nitrogen and fractured to obtain small fragments (0, 03 cm^3^) as described Orfila et al. [86]. Pericarp fragments were visualized by SEM as described above. Cell wall hydration analyses to check cell wall swelling capacity were performed following the protocols previously described by Orfila et al. [86].

A texture analyzer (TA-XT2 PLUS, Stable MicroSystems, Surrey, UK) was used to determine fruit compression stiffness and penetration mass as the force required to perforate the pericarp. To test the latter, the equatorial zone of the fruit was punctured in three different places, avoiding the septum, with a 4 mm probe. The probe's speed before and during the test was 10 mm per second and penetration mass was determined as the maximum peak of force reached expressed in grams. To determine the stiffness, fruits were compressed until reaching 5% of its diameter with a 12 cm diameter circular plane probe. Compression was analyzed and the probe's entry speed before and during of test was 2 mm per second.

## Supporting Information

Table S1 Schematic representation of gene expression analyses performed by quantitative RT-PCR in 35S:*TAGL1* and *TAGL1* RNAi plants as compared to wild type plants.
(0.09 MB DOC)

Table S2 Schematic representation of gene expression analyses performed by quantitative RT-PCR in sepals and fruits of *rin* and *nor* ripening mutants as compared to wild-type background (cv. Ailsa Craig, AC), as well as in *rin*-35S:*TAGL1* and *nor*-35S:*TAGL1* as compared to *rin* and *nor* mutants, respectively.
(0.06 MB DOC)

Table S3 Primers used for standard PCR analysis.
(0.04 MB DOC)

Table S4 Primers used for quantitative real-time PCR analyses.
(0.07 MB DOC)

Figure S1 The T-DNA insertion cosegregates with the *Alq* mutant phenotype.
(0.27 MB PPT)

Figure S2 Altered cell wall properties of *TAGL1* silenced fruits.
(0.56 MB PPT)
