# Supplementary material for: Functional Analysis of the Arlequin Mutant Corroborates the Essential Role of the ARLEQUIN/TAGL1 Gene during Reproductive Development of Tomato
Source: PLoS One. 2010 Dec 23;5(12):e14427. doi: 10.1371/journal.pone.0014427 (PMC3009712; doi:10.1371/journal.pone.0014427)
Supplement: Table S1 — Schematic representation of gene expression analyses performed by quantitative RT-PCR in 35S:TAGL1 and TAGL1 RNAi plants as compared to wild type plants. (0.09 MB DOC) [file pone.0014427.s001.doc]

**Table S1.** Schematic representation of gene expression analyses performed by quantitative RT-PCR in 35S:*TAGL1* and *TAGL1* RNAi plants as compared to wild type.

|  | **35S:*TAGL1* plants** | | | | | | | | | | ***TAGL1* RNAi plants** | | | | |
| --- | --- | --- | --- | --- | --- | --- | --- | --- | --- | --- | --- | --- | --- | --- | --- |
|  | Sepals | | | | | Carpels | | | | | **Carpels** | | | | |
|  | AD | IG | MG | BR | BR+8 | AD | IG | MG | BR | BR+8 | AD | IG | MG | BR | **BR+8** |
| LeDEF | ~ |  |  |  |  | ~ |  |  |  |  | ~ |  |  |  |  |
| MC | ~ |  |  |  |  | ~ | ~ | ~ | ~ | ~ | ~ | ~ | ~ | ~ | **~** |
| TAG1 | ~ | ~ | ~ | ~ | ~ | ~ |  |  |  |  | ~ |  |  |  |  |
| TAGL11 | ~ | ~ | ~ | ~ | ~ | ~ | ~ | ~ | ~ | ~ | ~ | ~ | ~ | ~ | **~** |
| TDR4 |  | ~ | ~ | ~ |  |  | ~ | ~ | ~ | ~ |  | ~ | ~ | ~ | **~** |
| ACS2 |  | ~ | ~ |  |  |  | ~ | ~ | ~ | ~ |  | ~ | ~ |  |  |
| ACS4 |  | ~ | ~ |  |  |  | ~ | ~ | ~ | ~ |  | ~ | ~ | ~ | **~** |
| ACO1 |  | ~ | ~ |  |  |  | ~ | ~ | ~ | ~ |  | ~ | ~ | ~ | **~** |
| NR |  | ~ | ~ |  |  |  | ~ | ~ | ~ | ~ |  | ~ | ~ | ~ | **~** |
| NOR |  | ~ | ~ |  |  |  | ~ | ~ | ~ | ~ |  | ~ | ~ | ~ | **~** |
| RIN |  | ~ | ~ |  |  |  | ~ | ~ | ~ | ~ |  | ~ | ~ | ~ | **~** |
| CNR |  | ~ | ~ | ~ |  |  | ~ | ~ |  |  |  | ~ |  |  |  |
| PSY |  | ~ | ~ |  |  |  | ~ | ~ | ~ | ~ |  | ~ | ~ |  |  |
| PE2 |  | ~ | ~ |  |  |  | ~ | ~ | ~ | ~ |  | ~ |  |  |  |
| PG |  | ~ | ~ |  |  |  | ~ | ~ | ~ | ~ |  | ~ | ~ |  |  |
| E4 |  | **~** | **~** |  |  |  | **~** | **~** | **~** | **~** |  | **~** | **~** |  |  |

Upward and downward arrows indicate up- and down-regulation, respectively, of the analysed genes. Compared to wild type plants changes of gene expression were indicated by one (2- to 10-fold), two (10- to 50-fold) or three (higher than 50-fold) arrows. Similar expression levels were indicated by ~ symbol. Ripening related genes were analyzed during four stages of fruit development and B-class *Le-DEF* gene was only analyzed in AD flowers. AD: anthesis day, IG: 2 cm immature green, MG: mature green, BR: breaker, BR+8: BR+8.
