# Supplementary material for: Functional Analysis of the Arlequin Mutant Corroborates the Essential Role of the ARLEQUIN/TAGL1 Gene during Reproductive Development of Tomato
Source: PLoS One. 2010 Dec 23;5(12):e14427. doi: 10.1371/journal.pone.0014427 (PMC3009712; doi:10.1371/journal.pone.0014427)
Supplement: Table S2 — Schematic representation of gene expression analyses performed by quantitative RT-PCR in sepals and fruits of rin and nor ripening mutants as compared to wild-type background (cv. Ailsa Craig, AC), as well as in rin-35S:TAGL1 and nor-35S:TAGL1 as compared to rin and nor mutants, respectively. (0.06 MB DOC) [file pone.0014427.s002.doc]

**Supplementary Table S2.** Schematic representation of gene expression analyses performed by quantitative RT-PCR in sepals and fruits (stage BR+8) of *rin* and *nor* ripening mutants as compared to wild-type background (cv. Ailsa Craig, AC), as well as in *rin*-35S:*TAGL1* and *nor*-35S:*TAGL1* as compared to *rin* and *nor* mutants, respectively.

| Gene | *rin* | | | | *nor* | | | |
| --- | --- | --- | --- | --- | --- | --- | --- | --- |
|  | sepals | | fruits | | sepals | | fruits | |
|  | *rin vs* AC | *rin*-35S:*TAGL1 vs rin* | *rin vs* AC | *rin*-35S:*TAGL1 vs rin* | *nor vs* AC | *nor*-35S:*TAGL1 vs nor* | *nor vs* AC | *nor*-35S:*TAGL1 vs nor* |
| *ALQ/TAGL1* |  |  | ~ |  | ~ |  | ~ |  |
| *TAG1* |  |  | ~ | ~ | ~ | ~ | ~ | ~ |
| *TDR4* | ~ | ~ |  | ~ | ~ | ~ |  | ~ |
| *ACS2* | ~ |  |  |  | ~ |  |  |  |
| *ACS4* | ~ | ~ |  |  | ~ | ~ |  |  |
| *ACO1* | ~ |  |  | ~ | ~ | ~ |  | ~ |
| *NR* | ~ | ~ |  | ~ | ~ | ~ |  | ~ |
| *NOR* | ~ |  | ~ | ~ |  |  |  |  |
| *RIN* |  |  |  |  | ~ |  |  | ~ |
| *CNR* | ~ |  |  | ~ | ~ |  |  |  |
| *PSY* | ~ |  |  |  | ~ |  |  |  |
| *PG* | ~ | ~ |  |  | ~ | ~ |  |  |
| *PE2* | ~ |  |  |  | ~ |  | ~ | ~ |
| *E4* | ~ | ~ |  |  | ~ |  |  |  |

Upward and downward arrows indicate up- and down-regulation, respectively, of the analysed genes. Compared to wild type plants changes of gene expression were indicated by one (2- to 10-fold), two (10- to 50-fold) or three (higher than 50-fold) arrows. Similar expression levels were indicated by ~ symbol.
