# Supplementary material for: Functional Analysis of the Arlequin Mutant Corroborates the Essential Role of the ARLEQUIN/TAGL1 Gene during Reproductive Development of Tomato
Source: PLoS One. 2010 Dec 23;5(12):e14427. doi: 10.1371/journal.pone.0014427 (PMC3009712; doi:10.1371/journal.pone.0014427)
Supplement: Table S3 — Primers used for standard PCR analysis. (0.04 MB DOC) [file pone.0014427.s003.doc]

**Table S3.** Primers used for standard PCR analysis.

| **Name** | **Primer sequence (5’ to 3’)** |
| --- | --- |
|  |  |
| GUS1 | ACTTTTCCCGGCAATAACATACG |
| GUS2 | TGATGCTCCATCACTTCCTGATT |
| GUS3 | CTGCCCAACCTTTCGGTATAAAG |
| 35SALQF | GGATCCTACCCAATCTTTGCTATATCGCC |
| 35SALQR | GGTACCAAACAGTTTAAATCTCAGTGGCAAT |
| RNAiALQF | TCTAGACTCGAGTACCCAATCTTTGCTATATCGCC |
| RNAiALQR | ATCGATGGTACCAACTGAGAAGACGACTCATCGAC |
|  |  |

Nucleotides introduced to generate restriction sites are underlined.
