# Supplementary material for: Functional Analysis of the Arlequin Mutant Corroborates the Essential Role of the ARLEQUIN/TAGL1 Gene during Reproductive Development of Tomato
Source: PLoS One. 2010 Dec 23;5(12):e14427. doi: 10.1371/journal.pone.0014427 (PMC3009712; doi:10.1371/journal.pone.0014427)
Supplement: Table S4 — Primers used for quantitative real-time PCR analyses. (0.07 MB DOC) [file pone.0014427.s004.doc]

**Supplementary Table S4.** Primers used for quantitative real-time PCR analyses.

| **Abbreviation** | **Gene** | **Accession**  **number** | **Forward (F) and reverse (R) primers** | **Expected size (bp)** |
| --- | --- | --- | --- | --- |
|  |  |  |  |  |
| *ALQ* | *ARLEQUIN* (*TAGL1*) | GU371906 | F-aaaagagggagattgagctgc  R-ctctacctctgctatctttgcg | 67 |
| *ALQ-pro* | *ARLEQUIN* (*TAGL1*) | GU371906 | F-gggaaggttccctacaatg  R-ggggttgctcatccatta | 76 |
| *CAD* | *Cinnamyl Alcohol Dehydrogenase* | AF146691 | F-cccctgttatgtgctggaat  R-ccaagaccaacaacaccaat | 92 |
| *LeCCR1* | *Cinnamoyl-CoA Reductase 1* | DQ019125 | F-gataacagcagcagcagagg  R-tgaggaagtaaacacaacgc | 52 |
| *CDKA1* | *Cyclin-dependent kinase A* | Y17225 | F-catagttttgcctggagtgg  R-agcctgcgtcatcaagaaat | 85 |
| *CycA1* | *Cyclin A* | AJ243451 | F-tactctactgtttttctctggct  R-tcagttgggatgttcttctca | 160 |
| *CycD3* | *Cyclin D* | AJ002590 | F-gaacatcaactccaccccc  R- gaaaactccaccaatcccat | 53 |
| *CNR* | *COLORLESS NON RIPENING* | DQ672601 | F-agttccattcaaagtctccaatag  R-cagatgaaatctgctacattgct | 82 |
| *E4* | *E4* | S44898 | F-agggtaatgatgtgggaaag  R-cttctaacgactcccttgcc | 87 |
| *ACO1* | *1-aminocyclopropane-1-carboxylic acid oxidase* | X58273 | F-acacattatttgctcttcttat  R-gcagcaaatcacaatctt | 68 |
| *ACS2* | *1-aminocyclopropane-1-carboxylic acid synthase* | AY326958 | F-taagtatgtaggatgaggagtat  R-tacgctaacaactatttctga | 46 |
| *ACS4* | *1-aminocyclopropane-1-carboxylic acid synthase* | X59146 | F-ataaactagattcgcccagg  R-ttgggcccgtgctttag | 43 |
| *LeDEF* | *Le-DEFICIENS* | AF052868 | F-acttacgccttcaacccaac  R-atcagagccacctccactgt | 62 |
| *MC* | *MACROCALIX* | AF448521 | F-tgaatggcaccagcaaacta  R-cctcccatatttaggcattga | 81 |
| *NOR* | *NON RIPENING* | AY573802 | F-tcgggttattggaaggctac  R-gccttttttaccccaacctt | 80 |
| *NR* | *NEVER RIPE* | U38666 | F-ggaaagggaaccactgtcac  R-tggtgacagattccgagttt | 50 |
| *PAL* | *Phenylalanine ammonia lyase* | M90692 | F-tgaagtgaaaaagatggtgg  R-ttccactttgaccccattac | 127 |
| *PE2* | *PECTIN ESTERASE 2* | X07910 | F-tgttggcgataaagctgaaa  R-accaccccacaaccataaac | 108 |
| *PG* | *POLYGALACTURONASE* | X14074 | F-agttgttggaggaggaggaa  R-ccaccatacttgtccattgc | 49 |
| *PSY* | *PHYTOENE SYNTHASE* | EF157835 | F-ggtgaagaggcaactgagat  R-ttcactcaacaagcccaaa | 85 |
| *RIN* | *RIPEINING INHIBITOR* | AF448522 | F-aggcatttgctaggtgagga  R-gttcaagctgttccaagtct | 67 |
| *TAG1* | Tomato *AGAMOUS* | L26295 | F-cttgatgccagggagttcat  R-atcgaattgctgaggtggag | 61 |
| *TAGL11* | *TAGL11* | AY098736 | F- attgttttctctactcgtggtc  R- tgcctttttgtatcgttctat | 81 |
| *TDR4* | *TDR4* | AY098732 | F-gtggcacagcaaaatcagtg  R-ccaaggtgaggagagtccag | 77 |
| *UBI3* | *UBIQUITIN3* | X58253 | F-cacacttcacttggtcttgcgt  R-tagtctttccggtgagagtcttca | 51 |
| *4CL* | *4-Coumarate:CoA ligase* | BF051818 | F- accaaaaggcgttcccataa  R- atgaaacagtggcaagacga | 115 |
|  |  |  |  |  |
