# Supplementary material for: Functional Analysis of the Arlequin Mutant Corroborates the Essential Role of the ARLEQUIN/TAGL1 Gene during Reproductive Development of Tomato
Source: PLoS One. 2010 Dec 23;5(12):e14427. doi: 10.1371/journal.pone.0014427 (PMC3009712; doi:10.1371/journal.pone.0014427)
Supplement: Figure S1 — The T-DNA insertion cosegregates with the Alq mutant phenotype. (0.27 MB PPT) [file pone.0014427.s005.ppt]

## Slide 1
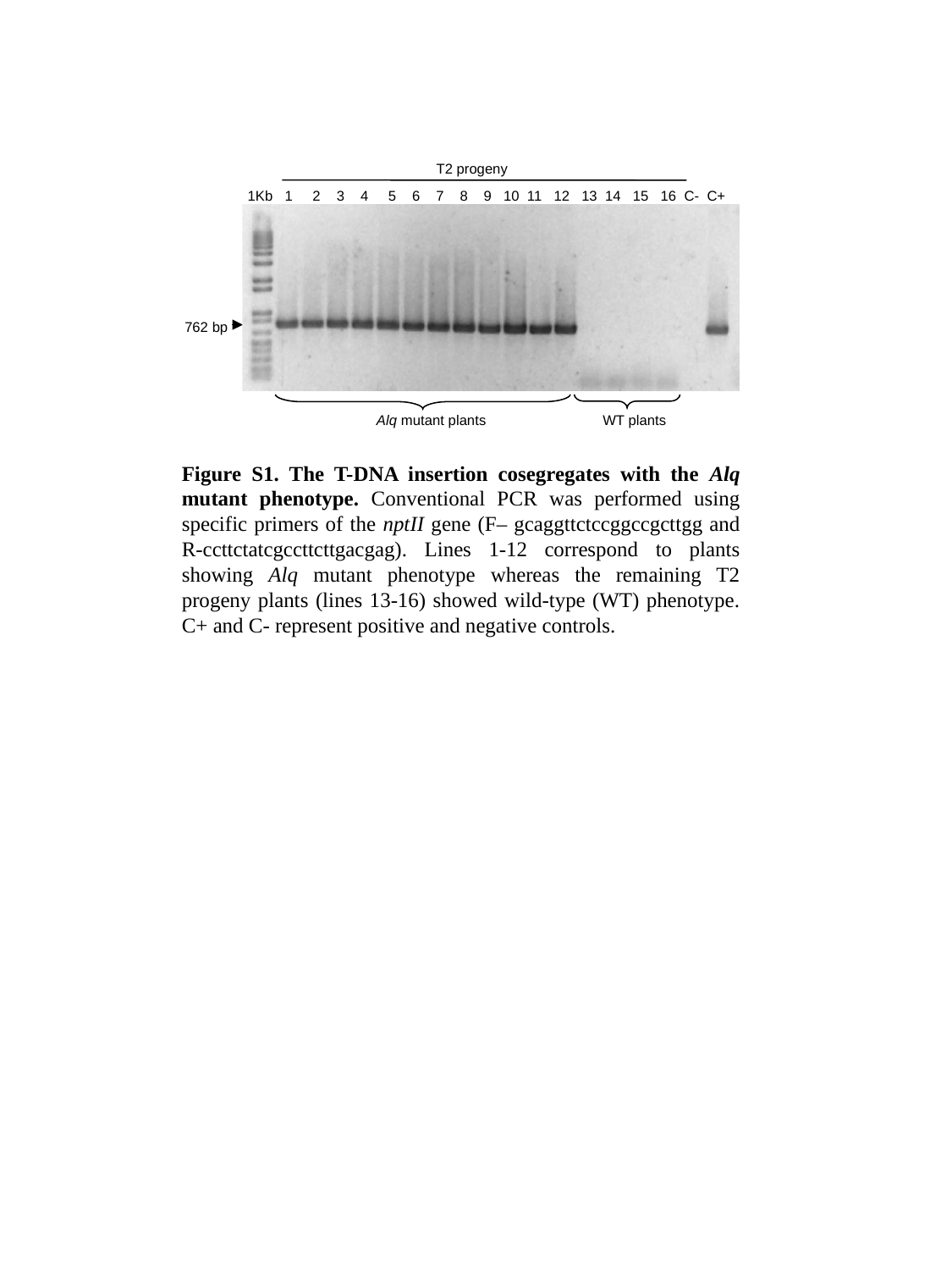

T2 progeny
 1Kb 1 2 3 4 5 6 7 8 9 10 11 12 13 14 15 16 C- C+
762 bp
Alq mutant plants WT plants
Figure S1. The T-DNA insertion cosegregates with the Alq mutant phenotype. Conventional PCR was performed using specific primers of the nptII gene (F– gcaggttctccggccgcttgg and R-ccttctatcgccttcttgacgag). Lines 1-12 correspond to plants showing Alq mutant phenotype whereas the remaining T2 progeny plants (lines 13-16) showed wild-type (WT) phenotype. C+ and C- represent positive and negative controls.
