# Supplementary material for: Functional Analysis of the Arlequin Mutant Corroborates the Essential Role of the ARLEQUIN/TAGL1 Gene during Reproductive Development of Tomato
Source: PLoS One. 2010 Dec 23;5(12):e14427. doi: 10.1371/journal.pone.0014427 (PMC3009712; doi:10.1371/journal.pone.0014427)
Supplement: Figure S2 — Altered cell wall properties of TAGL1 silenced fruits. (0.56 MB PPT) [file pone.0014427.s006.ppt]

## Slide 1
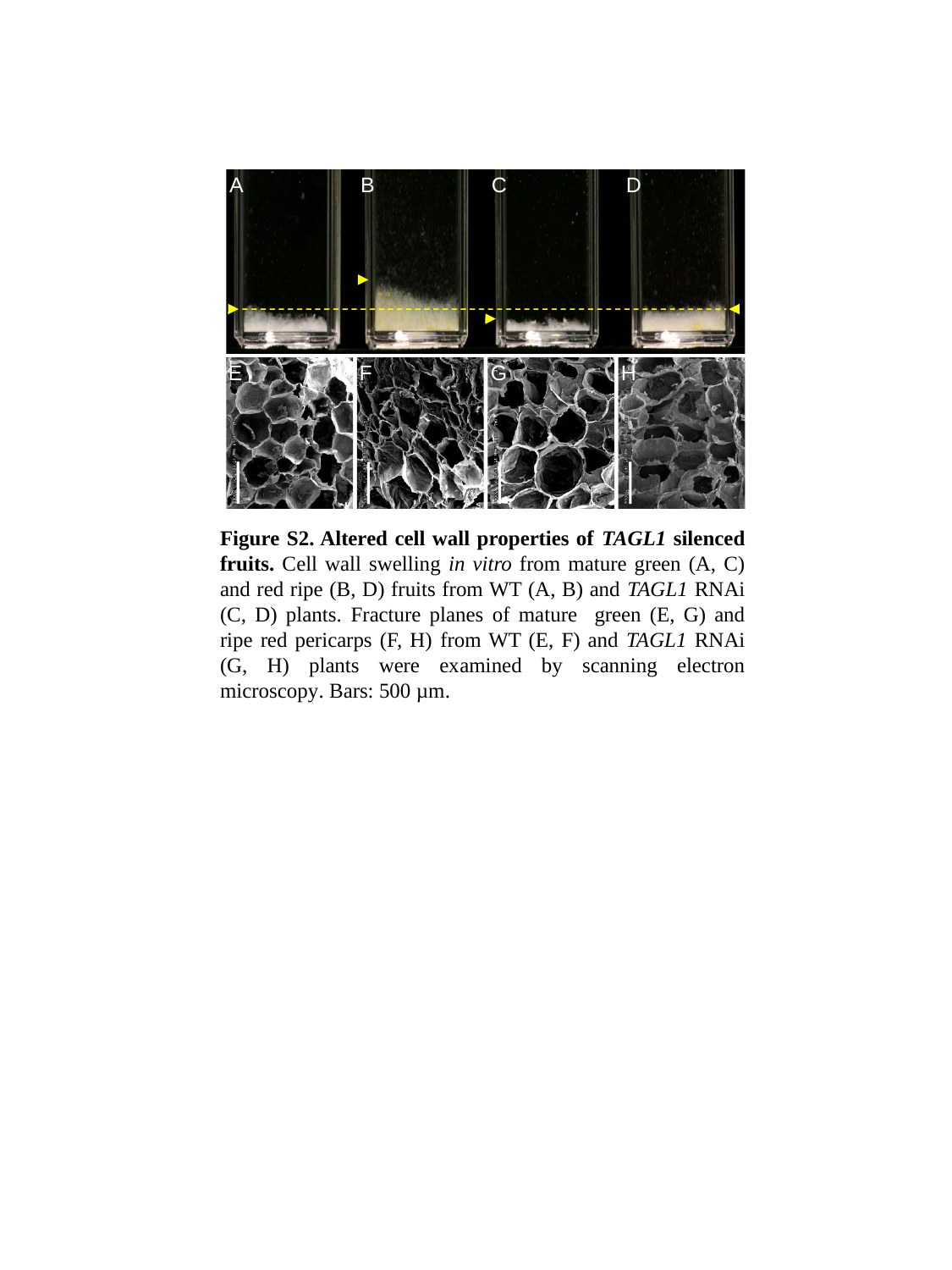

A
B
C
D
E
F
G
H
Figure S2. Altered cell wall properties of TAGL1 silenced fruits. Cell wall swelling in vitro from mature green (A, C) and red ripe (B, D) fruits from WT (A, B) and TAGL1 RNAi (C, D) plants. Fracture planes of mature green (E, G) and ripe red pericarps (F, H) from WT (E, F) and TAGL1 RNAi (G, H) plants were examined by scanning electron microscopy. Bars: 500 µm.
